# Xenomic networks variability and adaptation traits in wood decaying fungi

**DOI:** 10.1111/1751-7915.12015

**Published:** 2013-01-02

**Authors:** Mélanie Morel, Edgar Meux, Yann Mathieu, Anne Thuillier, Kamel Chibani, Luc Harvengt, Jean-Pierre Jacquot, Eric Gelhaye

**Affiliations:** 1 Université de Lorraine, IAM, UMR 1136IFR 110 EFABA, Vandoeuvre-lès-Nancy, F-54506, France; 2 INRA, IAM, UMR 1136Vandoeuvre-lès-Nancy, F-54506, France; 3 Laboratoire de biotechnologie, Pôle Biotechnologie et Sylviculture Avancée, FCBA, Campus Foret-Bois de Pierroton33610, Cestas, France

## Abstract

Fungal degradation of wood is mainly restricted to basidiomycetes, these organisms having developed complex oxidative and hydrolytic enzymatic systems. Besides these systems, wood-decaying fungi possess intracellular networks allowing them to deal with the myriad of potential toxic compounds resulting at least in part from wood degradation but also more generally from recalcitrant organic matter degradation. The members of the detoxification pathways constitute the xenome. Generally, they belong to multigenic families such as the cytochrome P450 monooxygenases and the glutathione transferases. Taking advantage of the recent release of numerous genomes of basidiomycetes, we show here that these multigenic families are extended and functionally related in wood-decaying fungi. Furthermore, we postulate that these rapidly evolving multigenic families could reflect the adaptation of these fungi to the diversity of their substrate and provide keys to understand their ecology. This is of particular importance for white biotechnology, this xenome being a putative target for improving degradation properties of these fungi in biomass valorization purposes.

## Introduction

Understanding the evolution of organisms and their adaptation to environmental constraints remains a major challenge for biologists. The recent development of sequencing technologies has led to a new science ‘Comparative genomics’. Gene copy number variation (CNV) has been observed from yeast (Carreto *et al*., 2008) to human (Redon *et al*., 2006). These CNVs result from duplication/deletion events. Duplication events can also be followed by functional diversification, this process being one of the most important mechanisms leading to new functions (Ohta, 1991; Force *et al*., 1999). The correlation between these genetic variations and phenotypic consequences remains often unclear. The most ambitious studies associating genetic and phenotypic variations are currently conducted in mammals in connection with the important implications in human health (Zhang *et al*., 2009; Yalcin *et al*., 2011). In contrast few studies have been devoted to fungal CNVs (Tirosh and Barkai, 2007; Liti *et al*., 2009; Ellison *et al*., 2011; Taylor, 2011). During the last years, several programmes have been launched to describe the functional genomic diversity of fungi, the relatively small size of their genome being compatible with the development of environmental genomic approaches (Sharpton *et al*., 2009; Martin, 2010; Neafsey *et al*., 2010; Ohm *et al*., 2010; Duplessis *et al*., 2011; Murat *et al*., 2011).

Fungi are major actors in global geochemical cycles, particularly in the carbon and nitrogen cycles, because of their involvement in organic matter recycling. Fungi play also major roles in different ecosystems through their symbiotic or pathogenic interactions with other organisms.

During the last few years, many sequencing projects have been devoted to wood-decaying fungi (Martinez *et al*., 2004; 2009; Eastwood *et al*., 2011), due to their potential applications in white biotechnology in general, and in bioenergy production from biomass in particular. Comparative genomic has confirmed the extraordinary diversity of enzymatic mechanisms developed by these fungi to degrade recalcitrant organic matter and in particular lignocellulose. Recent research has mainly focused on the oxidative systems secreted by white-rot and brown-rot fungi, and also on the myriad of fungal enzymes involved in the polysaccharide-degrading enzymes (Cazymes). Besides these extracellular systems, it appears that gene families involved in the detoxification pathways are particularly extended in wood-decaying fungi. These genes constitute the xenome, being defined as the biosystem responsible for the detection, transport and metabolism of xenobiotics (Edwards *et al*., 2005). Fungi as other organisms have to deal with potential toxic compounds resulting from organic matter degradation, secondary metabolism of antagonist organisms, and human activities. It is particularly true for wood-interacting fungi, which are able to degrade recalcitrant lignin and to cope with the myriad of complex compounds synthesized by plant species.

This article presents the fungal xenomic network with a comparative genomic analysis of two xenomic multigenic families found in wood decaying fungi. We have focused on the cytochrome P450 (CytP450) family, which is involved in the first step of compound modification mainly through oxidation reactions (phase I) and the glutathione transferase (GST) family, which belongs to the phase II conjugating enzymes and for which functional data are becoming available (Meux *et al*., 2011; Thuillier *et al*., 2011; Mathieu *et al*., 2012). In the literature, studying GST is usually justified because of the considerable interest of these enzymes in medicine, agriculture and analytical biotechnology. For example, in medicine, GSTs could be molecular targets for the design of new anticancer drugs. In agriculture, GSTs are exploited in the development of transgenic plants with increased resistance to biotic and abiotic stresses, or they could be used as biosensors for monitoring environmental pollutants, such as herbicides and insecticides (Chronopoulou and Labrou, 2009). In this review, we focus the analysis on fungal GSTs, which have been far less studied than their plant and mammals counterparts, postulating that these enzymes and more globally the xenome could be new targets of investigations in white biotechnology.

## Wood composition and degradation

Wood is mainly constituted of cellulose, a variety of hemicelluloses, lignin and to a lesser extent of secondary chemicals (wood extractives). Wood decomposition is a complex process, which, like litter degradation, involves biotic and abiotic factors (Cornwell *et al*., 2009; Weedon *et al*., 2009; Freschet *et al*., 2010). Numerous studies suggest that plant traits have predictable long-term effects on litter decomposition (Hobbie, 1996; Cornwell *et al*., 2008) and wood degradation (Cornwell *et al*., 2009; Weedon *et al*., 2009; Cornelissen *et al*., 2012). Among the factors, which lead to ‘afterlife effects’ on its degradation, wood chemical and anatomical traits are probably important (Cornwell *et al*., 2009). For instance, the decomposition of softwood is slower than hardwood in a given environment, suggesting that the chemical composition, which is highly variable between these wood types, could be an important factor governing wood decay (Weedon *et al*., 2009; Freschet *et al*., 2012). Wood is a complex structure, its composition depending both on the species considered and on environmental factors, which have influenced the tree growth (Chave *et al*., 2009). Wood susceptibility to fungal degradation is indeed governed at least in part, by its high content in lignin and other phenolic derivatives compounds.

The efficient fungal degradation of lignin is mainly restricted to specialized basidiomycetes. These latter are able to modify/decompose lignin through oxidative processes (see below), which could lead to aromatic radicals catalysing subsequent degradation and also formation of potential toxic molecules. Besides the well-known effect linked to lignin, secondary metabolites involved in plant defence could also play a major role in the durability of the various wood species (Chave *et al*., 2009; Freschet *et al*., 2012). The tree primary defence involves usually bark that provides a physical and chemical barrier against abiotic and biotic stresses. Besides these constitutive defences, induced systems are activated after injury or infection (Franceschi *et al*., 2005). For instance in conifers, induced defence concerns cell wall re-enforcements and production of secondary metabolites as terpenes, phenols, stilbenes, flavonoids and lignans (Ralph *et al*., 2006; Danielsson *et al*., 2011). These changes in chemical composition and their heterogeneity have potentially important ‘afterlife’ effects on the fungal efficiency in wood degradation.

## Fungal xenome: the phase I network

**Figure 1:**
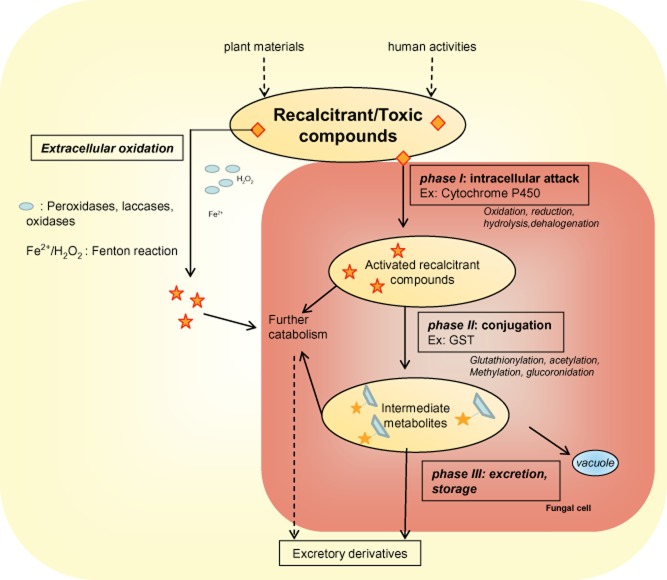
The fungal detoxification system defined as ‘xenome’.

Facing this large diversity of nutriments and potential toxic component sources, fungi, which mainly control wood decomposition in forest ecosystems, have also concomitantly evolved modified detoxification pathways, defining the xenome (Edwards *et al*., 2005). As in other eukaryotic organisms, the detoxification pathways dealing with recalcitrant/toxic compounds can be divided into three different steps in fungi, the first one corresponding to an activation of the molecule often through an oxidation stage, the second one to a conjugation stage, and the third one either to transport or to storage (Fig. 1). In this article, we will focus mainly on the oxidation and conjugation steps found in wood-decaying fungi through the determination of their xenomic gene content in relation to their environment.

### The extracellular network

In the case of wood decaying fungi, the first phase could be mediated by the extracellular oxidative network involved in lignin/wood modification. Wood-decaying fungi could be mainly divided into two main functional groups, brown and white rot, depending on their ability to degrade/mineralize lignin. The brown-rot fungi are able to modify lignin to gain access to cellulose and hemicellulose. The recent release of the genomes of *Postia placenta* and *Serpula lacrymans* has confirmed previous biochemical works demonstrating that this step is mainly mediated by non-enzymatic reactions in these fungi (Martinez *et al*., 2009; Eastwood *et al*., 2011). Brown-rot fungi use small phenolic compounds to reduce iron and produce hydrogen peroxide, prerequisite steps to form hydroxyl radicals via the Fenton reaction (Goodell *et al*., 1997; Eastwood *et al*., 2011). These radicals are involved in lignin modification and in polysaccharide degradation. In contrast, white-rot fungi are able to mineralize lignin, the oxidative degradation of the polymer being mainly mediated through the production of various oxidases and peroxidases (Hofrichter *et al*., 2010; Harms *et al*., 2011). Glycosyl hydrolases are mainly involved in polysaccharide degradation through hydrolytic processes (Martinez *et al*., 2004; 2009). Whatever the functional group considered, the oxidative stage exhibits low substrate specificity, explaining the well-studied potential of these fungi for bioremediation purposes (Harms *et al*., 2011). In particular, white-rot basidiomycetes are able to degrade a large panel of environmental pollutants (Asgher *et al*., 2008). Further information concerning the extracellular enzymatic networks involved in lignin breakdown can be obtained in recent reviews dealing with this topic (Sanchez, 2009; Wong, 2009; Hofrichter *et al*., 2010; Bugg *et al*., 2011; Hernandez-Ortega *et al*., 2012).

### The cytochrome P450 monooxygenases

The oxidative degradation of wood components leads to the formation of a variety of compounds with potential toxic effects depending on the initial substrate composition and also on the oxidative attack. To deal with these compounds, fungi possess a large number of CytP450. These enzymes, found in all life forms, are able to catalyse various reactions as hydroxylation, dealkylation, sulfoxidation etc. and could be involved in primary, secondary and detoxification metabolisms. These haem-thiolate proteins have first been discovered in liver microsomes (Omura and Sato, 1962) through their involvement in complex insertions of molecular oxygen into different substrates. General reviews on CytP450s have been published (Paine, 1995; Mizutani and Ohta, 2012; Nelson and Werck-Reichhart, 2011; Schuler, 2011; Ichinose, 2012).

In fungi as well as in other organisms, CytP450s have been shown to be involved in primary metabolism as CYP61 and CYP51 in ergosterol biosynthesis (Lepesheva and Waterman, 2007) and also in secondary metabolism as for instance in aflatoxin synthesis in aspergilli (Yu *et al*., 2004). Fungal CytP450s are also involved in detoxification pathways as shown in the basidiomycete *Phanerochaete chrysosporium* where various isoforms have been shown to be involved in polycyclic aromatic hydrocarbon oxidation (Syed *et al*., 2010; Hirosue *et al*., 2011). In basidiomycetes, the CytP450ome ranges from four (the pathogen *Cryptococcus neoformans*) to over 250 sequences (the brown-rot *P. placenta*), the number of isoforms apparently increasing with the ability to form mycorrhizae and to degrade plant litter (Cresnar and Petric, 2011). It is assumed that the high number of CytP450 isoforms found in wood decaying fungi could reflect the ability of these fungi to metabolize and mineralize aromatic compounds resulting from wood extracellular oxidation. The functional diversity of CytP450s has been investigated in particular in *P. chrysosporium*, where 154 CytP450-encoding genes have been identified in this white-rot basidiomycete (Martinez *et al*., 2004; Doddapaneni and Yadav, 2005; Chigu *et al*., 2010; Hirosue *et al*., 2011). Fourteen PcCytP450s isoforms are able to oxidize anthracene to anthroquinone and numerous isoforms are versatile enzymes accepting a broad range of substrates with varying three-dimensional structures (Hirosue *et al*., 2011). In contrast to CytP450 essential to primary metabolism, the isoforms involved in detoxification pathways are usually less specific (Cresnar and Petric, 2011). In *P. chrysosporium*, it is assumed that these versatile CytP450s have emerged from different ancestral genes due to extensive gene duplication events and intragenomic recombinations suggesting an evolution driven by the adaptation of the fungus to wood degradation (Doddapaneni and Yadav, 2005; Hirosue *et al*., 2011). From a general point of view mainly resulting from genomic data, it is now becoming obvious that the number and range of CytP450s within an individual organism reflect, to some extent, its lifestyle (Schuler, 2011). Transcriptional analyses confirm the role of various CytP450s in wood degradation/mineralization for both white-rot and brown-rot fungi. For instance, in the brown-rot *P. placenta*, where 230 genes are related to CytP450, differential induction of 15 encoding CytP450 genes was observed depending of the wood species incubated with the fungal culture (aspen versus pine) (Vanden Wymelenberg *et al*., 2010). In a similar way, in the white-rot *Phanerochaete carnosa*, 21 genes encoding CytP450s are found strongly regulated according to wood species (coniferous or deciduous wood) (MacDonald *et al*., 2011). All these data are in agreement with the following statement made by Hirosue and co-workers: ‘molecular evolution of basidiomycetous CytP450 has been, at least in part, vigorously driven by survival strategies to provide a superior metabolic system to degrade exogenous chemicals, presumably involving plant-related compounds including lignin and/or its derivatives’ (Hirosue *et al*., 2011).

## Fungal xenome: the phase II network

To cope with the compounds resulting from the activity of phase I enzymes, fungi possess large networks of phase II conjugative enzymes. It is widely admitted that conjugation enhances compound solubility and usually decreases their reactivity. The resulting conjugates could be excreted, further degraded or stored. These phase II enzymes catalyse the formation of sugar (glucoside, xyloside or glucuronide), sulfate, glutathione, acetyl or methyl conjugates. The different conjugates (and/or the different activities) have been detected during the degradation of various compounds in various fungal species (Sutherland *et al*., 1991; Bezalel *et al*., 1997; Hundt *et al*., 2000; Reddy and Gold, 2000; Myung *et al*., 2008; Campoy *et al*., 2009; Ning *et al*., 2010). Furthermore, as proposed for their plant counterparts, the different conjugates could be rapidly processed preventing their detection (Dixon *et al*., 2010). Conjugation enzymes exhibit a broad specificity accepting various substrates, which are structurally different. Despite this low specificity, transferases belong to multigenic families suggesting a specialization of the various isoforms. For instance, more than 100 putative glycosyltransferase-encoding genes are found in the brown-rot *P. placenta* (Eastwood *et al*., 2011). Nevertheless, such a high number of glycosyltransferases is not specific to wood-decaying basidiomycetes since similar contents are found in pathogenic/symbiotic ascomycetes (Klosterman *et al*., 2011), with nevertheless differential distribution within specific classes according to the way of life (Sprockett *et al*., 2011). Similarly, GSTs form a large ubiquitous multigenic family. The number of isoforms found in fungi range from six in *Saccharomyces cerevisiae* to more than 50, the highest number being found in the wood/litter decaying fungi (Morel *et al*., 2009). Besides these large families, the number of fungal arylamine *N*-acetyltransferases (NAT) ranged from one to five paralogues in fungi, NAT genes being lost in many species of the higher *Agaricomycotina* (Martins *et al*., 2010). Concerning sulfotransferases, few studies have been devoted to the diversity of these enzymes in fungi, but nevertheless this detoxification pathway seems to be restricted to ascomycetes.

## Fungal glutathione transferases

**Figure 2:**
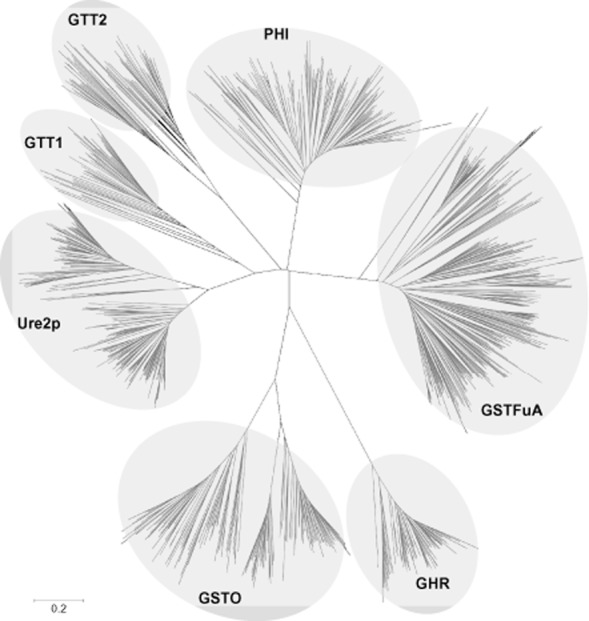
Phylogenetic tree of glutathione transferases from *Basidiomycetes*. Various subclasses could be distinguished: Ure2p, GSTFuA, Omega (GSTO), Glutathionyl Hydroquinone Reductase (GHR), Phi, GTT1 and GTT2. The sequences were retrieved from genomes available on the Joint Genome Institute (http://www.jgi.doe.gov/). Sequence alignments were done by clustalw and the tree was constructed with the neighbour-joining method in MEGA 5.0 software (Tamura *et al*., 2011). The robustness of the branches was assessed by the bootstrap method with 500 replications. The scale marker represents 0.2 substitutions per residue.

Soluble GSTs are usually dimeric enzymes, each monomer being composed of two domains. The N-terminal domain, which is more or less conserved, is involved in glutathione binding (G-site). The C-terminal domain is more variable and is involved in the binding of substrates (H-site). The nomenclature of cytosolic GSTs is based on amino acid sequence identity and at least eight classes have been described to date in fungi and named GTT1, GTT2, URE2p, MAK16, EFb1, Etherase-like recently renamed GSTFuA (Mathieu *et al*., 2012), GSTO and GHR (McGoldrick *et al*., 2005, Morel *et al*., 2009) (Fig. 2). Traditionally, two proteins belong to the same class if they share more than 40% identity and isoenzymes belonging to different classes share less than 20% (Hayes *et al*., 2005). Nevertheless, based on these primary sequence criteria only, many ‘non-canonical’ GST groups have emerged especially in bacteria and fungi, increasing the complexity of the GSTs classification. A few protein families are usually classified as GSTs (EF1Bγ, MAK16), but this is rather based on structural similarities, not on the existence of a glutathione-dependent activity. In addition, further immunologic, genetic, structural and functional investigations could reveal unexpected similarities between enzymes first listed in different groups (Meux *et al*., 2012). This emphasizes the challenge of GST classification.

It is largely admitted than the main activity of these enzymes is the catalysis of a glutathione adduct onto electrophilic molecules, the specificity of these enzymes remaining usually very weak. However, two classes of GSTs called omega (GSTO) and glutathionyl hydroquinone reductase (GHR) possess in their catalytic site a cysteinyl residue conferring to these proteins the ability to remove glutathione from various structurally different molecules (Garcera *et al*., 2006; Xun *et al*., 2010; Meux *et al*., 2011). These enzymes are highly versatile being able to switch activity when changing only one or few amino acids in the protein sequence (Mannervik *et al*., 2009). This property results in a large diversity of activities even within a single class, the enzymes being able to exhibit glutathionylation, deglutathionylation or peroxidase activity with various structurally different substrates.

**Figure 3:**
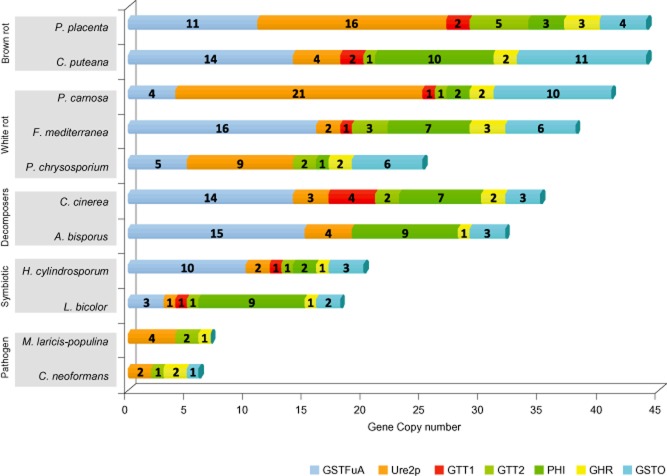
Organization of the GSTome in selected fungi. GST isoform numbers for each subclass are reported. The repartition has been done based on the sequences retrieved from the JGI coupled to phylogenetic analyses.

Taking advantage of the recent release of several basidiomycete genome sequences provided by the Joint Genome Institute (http://www.jgi.doe.gov), we describe in this review the occurrence of the main fungal cytosolic GSTs classes within fungi with various ways of life. Some selected species and their GST distribution are shown in Fig. 3. We have investigated the diversity of GSTs in saprophytic, symbiotic and pathogenic fungi (number of genes are given in supplementary data) and highlighted a huge diversity in term of GST number and repartition within the different classes. While the number of GST is higher in saprophytic fungi, no correlation could be established between the trophic mode of the fungi and their GST content.

### GTTs and phi classes

The GTT class has been defined for GlutaThione Transferase, since they correspond to the first characterized GST in *S. cerevisiae* (Choi *et al*., 1998). In yeast, it is composed of GTT1 and GTT2. Both proteins exhibit activity against classical GST substrates as 1-chloro-2,4-dinitrobenzene (CDNB). ScGTT2 also catalyses the formation of glutathione-Cd conjugates (Adamis *et al*., 2004), while ScGTT1 catalyses the reduction of hydroperoxides, in particular cumene hydroperoxide probably in the endoplasmic reticulum (Choi *et al*., 1998; Herrero *et al*., 2006). ScGTT1 and ScGTT2 have overlapping functions with glutaredoxins (ScGrx1 and ScGrx2) being involved in the response to oxidative stress (Mariani *et al*., 2008). Phylogenetic analyses concerning ascomycetes, zygomycetes and basidiomycetes have shown that GTT1 and GTT2 homologues cluster separately (Fig. 2) (Choi *et al*., 1998; McGoldrick *et al*., 2005; Morel *et al*., 2009). The number of GTT1 and GTT2 isoforms ranges from 0 to 4 for GTT1 and 0 to 5 for GTT2 (supplemental Table S1).

A separate cluster has been identified in the present phylogenetic analysis (Fig. 2). Since the amino acid sequences show similarity to the plant-related phi class, we named this new group GSTF for phi. In particular, the N-terminal part of some basidiomycete sequences exhibit around 75% of similarity with Poplar and Arabidopsis GSTFs. In fungi, the number of GSTF-encoding genes differs considerably depending on the genome considered, ranging from 0 to 10. The higher number (10 isoforms) is found in *Botryobasidium botryosum, Hydnomerulius pinastri, Coniophora puteana* and *Serpula lacrymans* (supplemental Table S1). A proteomic study revealed upregulation of *P. chrysosporium* phi isoform upon addition of exogenous application of vanillin, one of the key intermediates found during lignin degradation (Shimizu *et al*., 2005). However, since no isoform of GTT and phi has been characterized to date in basidiomycetes, their physiological role remains unclear.

### Ure2p class

GSTs belonging to this class have been found only in fungi and bacteria (except in *Stramenopiles* and *Dictyosteliida*). In *S. cerevisiae*, Ure2p acts both as a regulator of nitrogen catabolic repression, and as a stress-responsive protein (Coschigano and Magasanik, 1991; Blinder *et al*., 1996; Cooper, 2002). The protein has been well studied in yeast for its particularity to form prions (Wickner, 1994). The additional amino acids in the N-terminal part of the protein responsible for this property have been exclusively found in some yeast species, suggesting functional specialization of Ure2p in these organisms.

By analysing ascomycete and basidiomycete sequences, it has been shown that this class could be split into two subclasses called Ure2pA and Ure2pB. In each subclass, the sequences cluster according to the taxonomy suggesting a recent diversification of Ure2p sequences (Thuillier *et al*., 2011). The analysis of the fungal genomes reveals that only *Schizophyllum commune* does not possess any Ure2 sequence, suggesting an important role of this class among basidiomycetes (supplemental Table S1). Ure2pA is fungal specific and the number of isoforms is highly variable among species ranging from 0 in many species to 20 in *P. carnosa*. It is also interesting to note that species with the higher number of Ure2pA isoforms are saprophytic fungi. Ure2p4 and Ure2p6 of *P. chrysosporium* have been biochemically characterized (Thuillier *et al*., 2011). The corresponding genes are recent duplicates, the sequences being tandemly organized in the genome with 83% amino acid sequence identity. However the proteins do not exhibit the same activity pattern, Ure2p6 being much more active than Ure2p4 in both GST and thiol disulfide oxidoreductase activities.

Ure2pB is less expanded and more ubiquitous than Ure2pA (from one in most of the organisms to four sequences in *Agaricus bisporus, Coniophora puteana, Phlebia brevispora* and *Botryobasidium botryosom*) and is also present in bacterial genomes. Two isoforms (YfcG and YchU) have been characterized in *Escherichia coli* (Kanai *et al*., 2006). Both have thiol disulfide oxidoreductase activity and contrary to the yeast isoform, weak GST and peroxidase activities (Wadington *et al*., 2009; Stourman *et al*., 2011).

The expansion and diversification of Ure2p in *Agaricomycotina* could be linked to environmental conditions. Transcriptomic analyses revealed specific upregulation of some Ure2p-encoding genes in response to various wood species or aromatic compounds. As examples, *P. chrysosporium* Ure2p9 and Ure2p6 gene expression is induced in presence of aspen and pine respectively (Vanden Wymelenberg *et al*., 2011). In the same species Ure2p4 and Ure2p6 show specific expression after polycyclic aromatic compound treatments (Thuillier *et al*., 2011). Similarly, 8 Ure2p genes are upregulated by fir in *P. carnosa* (MacDonald *et al*., 2011).

### GSTFuA class

This class is only found in microbes (bacteria and fungi) (Morel *et al*., 2009). The proteins are related to Lig proteins of *Sphingobium* sp. SYK-6 (Masai *et al*., 1989). The Lig coding genes are organized in operons, which contains LigE, LigF and LigG. The corresponding proteins are involved in the cleavage of β-aryl ether linkages, which is the most abundant bond found in lignin. SpLigE and SpLigF are enantioselective GSTs involved in cleavage of β-aryl ether compounds, like guaiacylglycerol β-O-4-methylumbelliferone (GOU) to produce β-hydroxypropiovanillone and 4-methylumbelliferone *in vitro* (Otsuka *et al*., 2003). SpLigG is a glutathione lyase catalysing glutathione removal from the glutathione conjugate produced by SpLigF (Masai *et al*., 2003).

The number of isoforms is highly variable within and among fungal phyla and its distribution is not correlated to fungal taxonomy but rather to the trophic state of fungi. This class is indeed highly represented (between 4 and 19 isoforms) in wood interacting fungi (saprotrophic, litter-decomposing and necrotrophic fungi) whereas it is absent in the analysed biotrophic pathogens (supplemental Table S1). Interestingly ectomycorrhizal fungi also exhibit a high number of isoforms (from 3 to 10) in accordance with the observation that some of these fungi may live as facultative saprobes (Talbot *et al*., 2008). Unlike other fungal GSTs depicted here, the sequences cluster according to the organism and no specific subclass could be identified (Fig. 2).

### Cysteine-containing GSTs

Cysteine-containing GSTs are relatively ancient members of the cytosolic GST superfamily widespread in several kingdoms and phyla [Omega (O) in mammals, fungi and insect; *S*-glutathionyl hydroquinone reductase (GHR) in fungi and prokaryotes; Lambda (L) and DHAR in plants]. Although they exhibit the canonical thioredoxin fold, all these subclasses have a cysteine residue in their active site, where other GSTs exhibit a serine or a tyrosine. This cysteine changes dramatically the reactivity profiles of these enzymes compared with the classical GSTs. They do not catalyse conventional conjugation reactions and instead use GSH as a cofactor rather than co-substrate. Cysteine-containing GSTs have been described to catalyse glutathione-dependent reductions and thiol transferase reactions. They have been identified in all basidiomycete genomes studied here, whatever their trophic properties.

GSTOs are subdivided into two subclasses (II and III) and sequences are found in all symbiotic and saprophytic basidiomycete genomes with variability in their isoform number, while they were not detected in biotrophic fungi like *Melampsora larici populina, Puccinia graminis* or *Malassezia globosa* suggesting an evolution of the GST driven by the adaptation of the fungus to its habitat (supplemental Table S1). GHR sequences cluster separately and have been recently described as a new subclass of enzymes catalysing the removal of glutathione from hydroquinone conjugates (Xun *et al*., 2010; Belchik and Xun, 2011; Meux *et al*., 2011). One to four GHR isoforms have been detected in the studied fungal genomes, except for *M. globosa* and *P. graminis*, for which no GHR sequence has been found (supplemental Table S1).

In contrast to higher eukaryotes, fungal cysteine-containing GSTs have been poorly studied, and most of the investigations have focused on yeast. The *S. cerevisiae* genome encodes three proteins called GTO1, GTO2 and GTO3 that display similarities with human GSTO class (Garcera *et al*., 2006). These enzymes are active as thiol transferases, dehydroascorbate reductases and as dimethylarsinic reductases trough a monothiol mechanism (Garcera *et al*., 2006). Other experiments suggest a role of ScGTO1 in sulfur metabolism in the peroxisomes, which could be related to the redox regulation of the Str3 cystathionine β-lyase protein (Barreto *et al*., 2006). ScGTO2 is active towards several hydroquinone derivates (Lam *et al*., 2012) and its expression is strongly induced after exposure with stress agents, such as cadmium, hydroperoxide or diamide (Barreto *et al*., 2006), suggesting an involvement in oxidative stress response. Based on sequence similarity and functional data obtained with quinone derivatives, the yeast GSTOs isoforms could in fact be part of the glutathionyl hydroquinone reductases.

## A direct link between GSTome and P450ome

Comparative analysis of genome sequence data is an important tool to reveal adaptation of organisms to specific environments. Networks involved in drug metabolism and by extension in recalcitrant compound degradation/detoxification are particularly concerned by CNVs.

**Figure 4:**
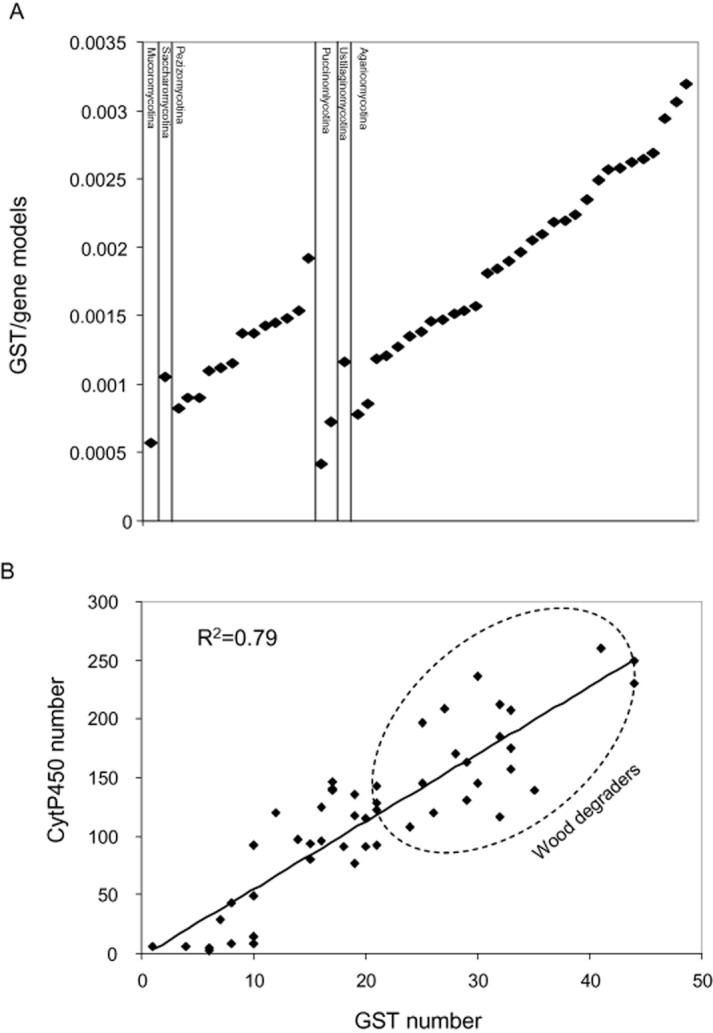
GSTome expansion in *Agaricomycotina*. A. Relative GSTome size of fungal species belonging to various phyla. The ratio between number of GST and number of gene models is reported. B. Relationship between GST and CytP450 copy numbers in the JGI available fungal genomes. Wood degraders exhibit the highest numbers of GST and CytP450 isoforms. Species names and data are given in supplemental Table S2.

Considering fungi from various phyla, a significant expansion of the total number of GST has been highlighted in *Agaricomycotina* (Fig. 4A and supplemental Table S2). This CNV is independent of the global number of gene models. This result can be correlated with a previous study performed on the CytP450 family (Cresnar and Petric, 2011). These authors showed that predominantly yeast-form fungi such as *Saccharomycotina* have a small P450ome, while mycorrhizal relationships and complex nutrient degradation mainly performed by *Agaricomycotina* seem to enhance the P450ome size. Based on this observation and the data obtained from the Fungal Cytochrome P450 database (http://p450.riceblast.snu.ac.kr/species.php), a direct correlation between P450ome and GSTome contents can be observed suggesting a close link between phase I and phase II detoxification processes (Fig. 4B). However, this correlation with CytP450 does not exist when considering the GST classes individually. This suggests that compensatory effects between GST gene copy numbers could exist to relay the first detoxification step. Both protein families are largely expanded in wood-interacting fungi. For instance, the symbiotic *Laccaria bicolor* exhibits a weak ability to degrade recalcitrant organic matter in correlation with relatively restricted xenomic content. In contrast, *Paxillus involutus*, another symbiotic basidiomycete, possesses an extracellular oxidative system (Rineau *et al*., 2012) and also an expanded xenome. The most expanded xenomes are found in fungi interacting with wood as the necrotrophic *Heterobasidion annosum* and of course the white and brown-rot fungi.

As proposed for CytP450s, the diversification of GSTs is also recent and could be driven by the adaptation of the fungi to their environment. An analysis of the rapid divergent genes associated in particular with duplication/deletion events has been conducted in various isolated strains of the symbiotic fungus *P. involutus* (Le Quere *et al*., 2006). Among the identified CNVs, the authors reported at least three CytP450 and one GST-encoding genes demonstrating that the variation in gene copy number occurring in this fungus at the isolate level concerns these gene families (Hedh *et al*., 2009). In other organisms, CytP450s and GSTs are also concerned by CNVs as shown for instance for the human cytochrome P450 2D6, this polymorphism being associated with drug metabolizing systems (He *et al*., 2011). In a similar way, the human genome encodes 17 soluble GSTs, two of them missing in some individuals (Mannervik, 2012). All these data agree with a rapid evolution of the xenomic network driven by the molecular environment of the organisms.

### Coexpression of P450ome and GSTome

**Figure 5:**
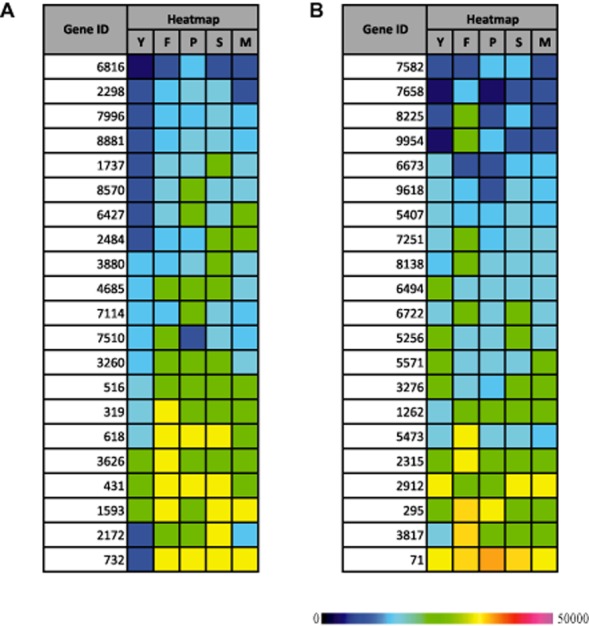
Heat maps for selected *P. carnosa* transcripts coding for CytP450 (A) and GSTs (B), during growth on YMPG (Y) or medium containing wood from fir (F), pine (P), spruce (S) or maple (M), as determined by mRNA-Seq. The colour scale represents the reads per kilobase of gene model per million mapped reads. The figure has been adapted from MacDonald and colleagues (2011).

The direct correlation between the P450ome and GSTome was also investigated using the growing number of transcriptional studies. For example, specific CytP450-encoding genes found in *P. chrysosporium* are induced in presence of polycyclic aromatic hydrocarbons (Syed *et al*., 2010) and it is also the case for two Ure2p isoforms (Thuillier *et al*., 2011). In the same fungus, nonylphenol induces strongly three CytP450 and a Ure2p-encoding genes (Subramanian and Yadav, 2009). In the context of wood degradation, induction of different CytP450s-encoding genes has been observed in *P. chrysosporium* and *P. placenta* in a wood species-dependent manner (Vanden Wymelenberg *et al*., 2011) and *P. carnosa* (MacDonald *et al*., 2011). In *P. carnosa*, heat maps revealed variable expression of CytP450 and GST genes during growth on fir, pine, spruce or maple (Fig. 5 adapted from MacDonald *et al*., 2011). Some transcripts have been found to be abundant in all wood degrading conditions, while others seem to accumulate in specific ones. As an example CytP450 (Gene ID 516) and GST (Gene ID 1262) exhibit the same transcript profile, i.e. an induction by all wood substrates. Similarly, CytP450 (Gene ID 3626) and GST (Gene ID 2315) are both induced specifically by fir (Fig. 5). Concomitant analysis of CytP450 and GST expression could be a starting point to establish a functional link between isoforms.

### Functional links between GSTome and P450ome

The direct correlation observed between the CytP450 and GST contents raises the question of the functions of the different isoforms. CytP450 are usually involved in the catalysis of redox reactions leading to the production of oxidized products. These oxidation steps could be associated with biosynthetic pathways in particular of secondary metabolites. The activities of CytP450 could lead to the formation of potential GST substrates containing electrophilic and unstable centres such as epoxides. In plants, the xenobiotic glutathionylation is relatively well documented, the GSH-conjugates being imported into the vacuole through ABC transporters before degradation (Grzam *et al*., 2006; 2007; Rea, 2007). A non-vacuolar degradation pathway of GSH conjugates has been also shown in *Arabidopsis thaliana* (Blum *et al*., 2007). The catabolism of glutathione conjugates has been studied in yeast showing the potential involvement of vacuolar serine carboxypeptidases (Wunschmann *et al*., 2010). In fungi, besides the role of GTT in peroxide detoxification (Herrero *et al*., 2006) the functions of the different GST remain essentially elusive. As stated for plant GSTs, these proteins could be involved of course in glutathione conjugation but also in the transport of unstable metabolic intermediates using GSH as a stabilizing partner (Dixon *et al*., 2010). For instance, exposure to HAP in different fungi leads both to the accumulation of toxic compounds in intracellular vesicles and to specific GST gene induction supporting the hypothesis of their involvement in intracellular transport (Verdin *et al*., 2005; Thuillier *et al*., 2011; Thion *et al*., 2012).

Besides the formation of glutathionylated conjugates, different isoforms are able to catalyse the reverse reactions. This is particularly true for cysteine containing GSTs as GSTOs but also for several isoforms of Ure2p. As stated before, the activities of CytP450 lead in numerous cases to unstable intermediates, which could react spontaneously with the intracellular reduced glutathione (present at a millimolar range in fungi). In addition to the potential mechanistic role of GSTs in deglutathionylation (thiol transferase activity), these enzymes could also restore the initial compound allowing the pursuit of the following metabolic/catabolic steps.

## Xenomic genes and neo-functionalization

Enzymes involved in detoxification pathways are usually able to accept various substrates exhibiting a broad specificity. Nevertheless, the expansion of xenomic genes found in particular in wood decaying fungi suggests a more specific function for each isoform. In both cases (CytP450s and GSTs), small changes in substrate binding sites alter strongly the specificity of the resulting enzymes. The evolution of these protein superfamilies is complex, involving gene duplication, followed by mutations of the primary structure.

Concerning fungal GSTs, few biochemical data are available in particular for basidiomycetes. An illustration is the recent evolution of PcUre2p4 and PcUre2p6 isoforms, which have gained or lost functions in spite of a very similar sequence (Thuillier *et al*., 2011). Another example concerns GSTFuA isoforms. Most of the sequences exhibit a serine at the putative active site, which is known to be involved in glutathionylation activity. However some isoforms possess a glycine or an alanine instead of a serine suggesting functional divergence. Site-directed mutagenesis performed on one *P. chrysosporium* isoform has demonstrated that changing this serinyl residue into an alanine alters strongly the activity of the resulting protein conferring for instance a new thiol transferase activity to the mutated protein. Similarly, a functionomic approach resulted in highlighting the versatility of CytP450s from *P. chrysosporium* by screening a wide variety of compounds as substrates (Hirosue *et al*., 2011).

Fungal GSTs and CytP450s are thus very interesting models to understand protein evolution through promiscuous catalytic activities, using for instance the mutational approaches developed by Mannervik and co-workers for the characterization of mammals GSTs (Mannervik, 2012). On the other hand, distinct structural classes of fungal GSTs exhibit similar patterns of activities. It is the case for instance for some GSTFuA and Ure2p isoforms of *P. chrysosporium* suggesting a potential functional convergence between these classes. A similar convergence has been also observed for versatile CytP450, which are able to catalyse similar reactions without significant sequence similarity (Hirosue *et al*., 2011).

## Xenomic genes and fungal adaptation

Globally, the xenome, described in this article through CytP450ome and GSTome analysis, is a rapidly evolving network. It is particularly true in wood decaying fungi where both families are largely expanded. The factors driving this evolution remain unclear but we postulate that these expansions should be correlated with the extraordinary ability of these fungi to deal with (and to catabolize in numerous cases) recalcitrant compounds. As for other organisms, fungal genomes display duplication regularly, and only duplications that increase fitness are selected and persist (Taylor, 2011). Concerning CytP450, a well-documented example concerns the co-evolution of plant and insect CytP450omes (Schuler, 2011). Plant CytP450s are involved in secondary metabolism leading to the production of compounds involved in plant protection, whereas insect CytP450 are mainly responsible for the catabolism of these toxic compounds. We postulate that similar relationships between trees and wood-degrading fungi could at least in part govern the evolution of xenomes of wood-degrading fungi. The wood susceptibility to degradation is complex and depends of many factors. Among them, the wood chemical composition plays a predominant role, depending of course on the considered tree species, but also on the considered tree part and on environmental factors (biotic and abiotic) which influence the tree life.

On the other hand, wood decomposers are more or less specific for wood species and act differentially along the degradation process (Bässler *et al*., 2012). Fungal decay of wood could thus be seen as a succession of heterotrophic events mediated by various fungal populations, which have adapted their xenome to various wood features. Besides the ability to degrade/modify wood components and in particular lignin, through extracellular oxidative and hydrolytic networks, interactions of wood-decaying fungi with their environment could be also considered through their xenomic contents.

Fungal CytP450s and GSTs are involved in numerous catabolic pathways allowing wood-decaying fungi to cope with their specific way of life. The xenome could also be a major factor governing the ability of fungi to colonize specific habitats. A recent comparative genomic analysis revealed that *P. carnosa* is highly enriched with genes encoding CytP450 compared with *P. chrysosporium* (nearly double) (Suzuki *et al*., 2012). *P. carnosa* has been isolated almost exclusively from softwood, which is the most recalcitrant biomass resources, while *P. chrysosporium* was mainly isolated from hardwoods. Comparative growth studies on model compounds and chemical analyses of decomposed wood components showed greater tolerance of *P. carnosa* than *P. chrysosporium* to various substrates including coniferous heartwood. The significant expansion of CytP450s and GSTs in *P. carnosa* could thus be correlated with its ability to cope with the recalcitrant softwood compounds.

## Conclusion

Numerous studies are devoted to the description and the characterization of extracellular enzymes involved in lignocellulose breakdown. In wood decaying fungi, the oxidative and hydrolytic systems are particularly extended demonstrating the adaptation of these fungi to the diversity of their substrates. This adaptation occurs at least in part through the development of multigenic families (Cazymes or transporters for instance). From the recent release of basidiomycete genomes, we postulate that xenomic genes could also reflect the adaptation of these fungi to their way of life and could be used as markers to correlate genomic to phenotypic variations in a context of environmental adaptation. In fact, behind the versatility of the enzymes described in this article, we postulate that some specificity drive the extension of these families. Since wood chemical composition is a major factor governing its own microbial degradation, it could play a major role in xenome variability. The next challenge will be to connect this xenomic variability to the ecology of these wood-decaying fungi to better understand how they can both degrade complex compounds and resist to the associated toxic molecules. Moreover, combining data concerning both GSTome and CytP450ome could have promising implications in biotechnological purposes such as wood preservation, fungicide resistance or biomass valorization. The yield of enzymatic hydrolysis being increased by pre-treatment with ligninolytic fungi (Giles *et al*., 2011), the selection of strains with better degrading capabilities and resistance mechanisms to toxic molecules will provide potentially economic, social and health benefits.

## Conflict of interest

None declared.

## Supporting information

Table S1 GST isoform number in basidiomycetes and repartition within the various subclasses. Sequences were retrieved by BLAST search from the JGI database and the repartition was done by a phylogenetic analysis using MEGA5.

Table S2 Total number of GST and CytP450 in various species from *Agaricomycotina, Ustilagomycotina, Pucciniomycotina, Pezizomycotina, Saccharomycotina* and *Mucoromycotina*.
