# Supplementary material for: Xenomic networks variability and adaptation traits in wood decaying fungi
Source: Microb Biotechnol. 2013 Jan 2;6(3):248–63. doi: 10.1111/1751-7915.12015 (PMC3815920; doi:10.1111/1751-7915.12015)
Supplement: Table S1 — GST isoform number in basidiomycetes and repartition within the various subclasses. Sequences were retrieved by BLAST search from the JGI database and the repartition was done by a phylogenetic analysis using MEGA5. [file mbt0006-0248-sd1.doc]

Supplemental Table 1: GST isoform number in basidiomycetes and repartition within the various subclasses. Sequences were retrieved by BLAST search from the JGI database and the repartition was done by a phylogenetic analysis using MEGA5.
