# Supplementary material for: Xenomic networks variability and adaptation traits in wood decaying fungi
Source: Microb Biotechnol. 2013 Jan 2;6(3):248–63. doi: 10.1111/1751-7915.12015 (PMC3815920; doi:10.1111/1751-7915.12015)
Supplement: Table S2 — Total number of GST and CytP450 in various species from Agaricomycotina, Ustilagomycotina, Pucciniomycotina, Pezizomycotina, Saccharomycotina and Mucoromycotina. [file mbt0006-0248-sd2.doc]

Supplemental Table 2: Total number of GST and CytP450 in various species from Agaricomycotina, Ustilagomycotina, Pucciniomycotina, Pezizomycotina, Saccharomycotina and Mucoromycotina.
